# Thymol, the New Antiseptic

**Published:** 1878-08

**Authors:** 


Thymol, the New Antiseptic.—A rival to carbolic acid has certainly been discovered in thymol, the essential ingredient of oil of thyme, which is prepared either from the oil of thyme itself or by distilling the seeds of *Ptychotis ajowan*, an East Indian umbellifer, which contain from five to six per cent. of their weight of this body. This substance was first discovered by Neumann in 1719, but its antiseptic properties were first definitely recognized by Dr. L. Lewin, of Berlin, in 1875. Lewin, who worked in Professor Liebreich's laboratory, showed experimentally that solutions containing one part thymol per 1000 absolutely arrested saccharine fermentation; and that they powerfully retarded lactic fermentation, and checked various processes of decomposition, even when used in relatively small quantities. Lewin also first pointed out the comparative harmlessness of thymol internally administered; the absence of digestive disturbance after taking it, and its effects in checking abnormal fermentation in the stomach. He further directed public attention to the probable future of the drug as an antiseptic. Husemann's experiments, which were chiefly made on rabbits and frogs, went to show that thymol is ten times less poisonous to the organism than carbolic acid, and that hence in the quantities ordinarily used for antiseptic purposes, it may be considered entirely innocuous. He further showed that thymol is a far more powerful antiseptic than carbolic acid, that its local application to the skin, either as such, or in saturated solutions, had no irritant effect whatever, and that in animals poisoned by excessive doses, gastric erosions never occurred, as they do in carbolic acid poisoning, but that, on the other hand, nephritis, with albuminous urine, and extensive fatty disintegration of the liver, are nearly constant phenomena in these cases.

Professor Volkmann, of Halle, has introduced it into his clinic, and Dr. Hans Ranke reports the results as striking in the extreme. In the main, the general features of Lister's antiseptic dressings were retained by Ranke, thymol being substituted for carbolic acid. Since thymol is not entirely soluble in water in proportion of 1 to 1000, the following formula was, after the first few trials, exclusively used for antiseptic purposes: Thymol, 1 gramme; alcohol, 10; glycerine, 20; water, 1000 grammes. This "thymol solution," as it may be called for brevity's sake, has no corrosive action on instruments immersed in it, and in this respect is superior to solutions of carbolic, and still more salycilic acid. It causes, however, when sprayed over the hands of the operator, a lively sensation of burning, accompanied with redness of the skin, but otherwise has no irritant qualities. Anesthesia of the skin and epidermic desquamation, both of which are liable to occur under the use of carbolic acid, were never once observed in the case of thymol, nor did it exert any irritant action on the respiratory organs.—*Medical Times and Gazette.*

